# Cerebral Kounis Syndrome—A Rare Case Report of Cerebral Vasospasm Following Anaphylaxis

**DOI:** 10.5811/cpcem.50776

**Published:** 2026-06-27

**Authors:** Mohd Helmie Ismail, Sadesvaran Muniandy, Muhammad Shafiq Azmi

**Affiliations:** * Sultan Ahmad Shah Medical Centre @IIUM, Department of Emergency and Trauma, Kuantan, Pahang, Malaysia; † International Islamic University Malaysia, Kulliyyah of Medicine Kuantan, Department of Emergency Medicine, Pahang, Malaysia; ‡ Hospital Kemaman, Emergency and Trauma Department, Kemaman, Terengganu, Malaysia

**Keywords:** cerebral vasospasm, anaphylaxis, Kounis syndrome, case report

## Abstract

**Introduction:**

Kounis syndrome describes anaphylaxis-induced coronary vasospasm and is often misdiagnosed as acute coronary syndrome. We report a similar phenomenon of cerebral vasospasm following anaphylaxis, a rare and under-recognized mimic of stroke.

**Case Report:**

A previously healthy 45-year-old gentleman developed sudden right-sided hemiparesis and dysarthria while working outdoors, accompanied by generalized pruritus, rash, presyncope, dyspnea, palpitation, and abdominal pain. He received prompt intramuscular epinephrine and other anti-inflammatory agents, resulting in rapid symptom resolution and complete recovery in the emergency department. Computed tomography of the brain and other studies were unremarkable. This case underscores a rare neurological manifestation of anaphylaxis.

**Conclusion:**

Cerebral vasospasm is an unusual sequela of anaphylaxis that may lead to a diagnostic dilemma. Prompt administration of epinephrine is crucial for recovery. This case highlights the association between hypersensitivity reactions and cerebrovascular events, emphasizing the need for early recognition and timely intervention.

## INTRODUCTION

Anaphylaxis is a severe, life-threatening allergic reaction that can lead to rapid cardiovascular, respiratory, and cutaneous complications. It is estimated to affect 1.6–5.1% of the global population, with insect stings, foods, and medications being the most common triggers.^1^ Anaphylaxis is typically associated with hypotension, bronchospasm, and urticaria. However, it can also cause rare neurological complications and even present similarly to stroke or transient ischemic attack. While this has been documented, it remains under-recognized in clinical practice.^2^–^6^

Kounis syndrome, a condition in which an allergic reaction triggers coronary artery vasospasm, has been expanded to include cerebral vasculature involvement, suggesting that anaphylaxis may lead to stroke mimics.^2^,^7^ This process is attributable to local vasospasm, as it involves abnormal mast-cell activation or systemic macrocytosis, resulting in the release of acute inflammatory mediators during the reaction and affecting multiple arterial beds, including the cerebral circulation.^7^ We report a unique instance of a cerebral vasospasm following an anaphylactic reaction in a previously healthy 45-year-old male. This case highlights the rare but significant neurological consequences of anaphylaxis and contributes to the growing body of literature on the association between allergic reactions and cerebrovascular events.

## CASE REPORT

A previously healthy 45-year-old male with no previous history of allergy presented to the emergency department (ED) after a sudden and severe allergic reaction. He had been cutting trees in a nearby forest when he suddenly experienced intense itchiness over his entire body, followed by the appearance of a rash on his extremities. As his symptoms progressed, he developed lightheadedness, shortness of breath, palpitations, colicky abdominal pain, right-sided hemiparesis, and slurred speech. Upon arrival at the ED, the patient was alert and conscious but speaking with a slurred voice. His vital signs were recorded as follows: blood pressure, 154/96 millimeters of mercury; heart rate, 102 beats per minute; respiratory rate 18 breaths per minute; and temperature, 37 °Celsius. Neurological examination revealed right-sided weakness (3/5) in both the upper and lower limbs, normal tone, normal reflexes, and an equivocal Babinski response. The left side of his body was unaffected. Cranial nerve examination showed normal findings, and there was a wheal rash on his trunk—a well-defined erythematous rash.

**Image 1:**
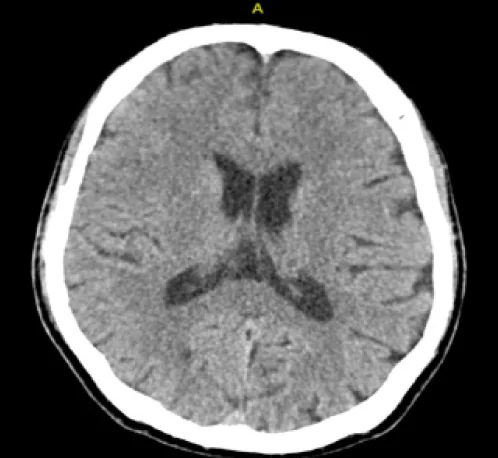
Computed topography of the brain shows no evidence of infarct with preserved grey-white matter differentiation.

**Image 2:**
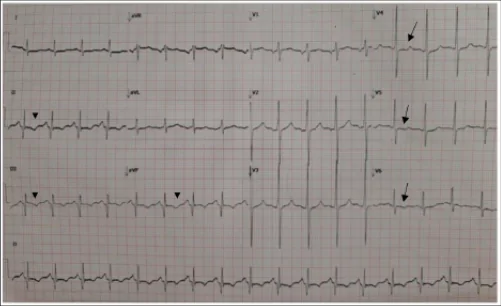
Electrocardiogram showing sinus tachycardia with ST-segment depression over leads V4–V6 (arrows) and T-wave inversion at II, III and aVF (arrowheads).

Given the constellation of symptoms, the patient was immediately treated for anaphylaxis. He was administered intramuscular epinephrine, 0.5 milligram (mg); intravenous (IV) hydrocortisone, 200 mg; IV chlorpheniramine, 10 mg; and IV ranitidine, 50 mg. Computed tomography of the brain revealed no evidence of acute infarction, with preserved grey-white matter differentiation (Image 1). Other blood investigations, including serum electrolytes, were within normal ranges, and the serum glucose level was 5.7 millimoles per liter (mmol/L) (reference range: 4.1–7.8 mmol/L). Results of the electrocardiogram performed is shown in Image 2. Within minutes of treatment, the patient experienced marked improvement. His right-sided hemiparesis resolved, and he was able to speak normally without slurring. By the time of observation, his symptoms had completely resolved. Despite the positive response to treatment, he opted to discharge himself against medical advice and was scheduled for a follow-up appointment at a specialist clinic.

*CPC-EM Capsule*

What do we already know about this clinical entity?

*Kounis syndrome is an allergy-induced vasospasm, usually affecting coronary vessels.*

What makes this presentation of disease reportable?

*Cerebral vasospasm presented as a stroke mimic during anaphylaxis, resolving rapidly with intramuscular epinephrine.*

What is the major learning point?

*Anaphylaxis can cause cerebral vasospasm and present as a stroke mimic. Neurological deficits may occur, regardless of blood pressure, and require prompt epinephrine.*

How might this improve emergency medicine practice?

*Clinicians should screen stroke mimics for allergic signs. Prompt epinephrine and anti-inflammatory agents can reverse vasospasm and prevent ischemic damage.*

## DISCUSSION

Anaphylaxis is a severe, life-threatening systemic hypersensitivity reaction that predominantly affects the respiratory and cardiovascular systems. Typically, anaphylaxis is associated with symptoms such as hypotension, bronchospasm, urticaria, and angioedema. Neurological complications, while possible, are exceedingly rare in anaphylaxis.^1^ In this case, the diagnosis was debatable given the broad range of differential diagnoses suggested by the initial presentation, including ischemic stroke, migraine, hypoglycemia, and vasovagal collapse, compounded by uncertainty regarding the triggering allergen. Nevertheless, the presence of early allergic manifestations such as flushing, generalized pruritus, dizziness, shortness of breath, and cutaneous involvement, along with stroke-like symptoms, led to high index of suspicion of anaphylaxis as it occurs rapidly and involves multiple organs. This in turn triggers the clinician to administer anti-inflammatory agents. While anaphylaxis normally occurs without circulatory shock or breathing complications,^1^ in this case, it could have been argued as the blood pressure was not hypotensive; however, a recent study found that even in anaphylactic shock, the cerebral blood flow will decrease more than the expected peripheral arterial hypotension.^2^,^8^ Thus, it is possible that severe impairment of cerebral blood flow can occur, which may not be explained by the level of peripheral arterial hypotension.

While the triggering cause of the allergic reaction was not identified, it is believed to have been induced by a lipid soluble substance because immunoglobulin E (IgE) cannot cross the blood brain barrier even though the mast cells are ubiquitous.^4^ The likely allergen was a neurotoxic hymenoptera sting sustained in a forested area.^5^,^6^ Another possibility is that it was induced by nonIgE mediated hypersensitivity. It has also been theorised that previous exposure to the allergen, such as pollen in the forest, could sensitize the mast cells in the brain, allowing the degranulated mast cells to infiltrate into the affected cerebral vessels even before the initial event occurs.^9^,^10^ This could explain why anaphylaxis occurs at any age, but with broadening clinical manifestations, as it is caused by a wide spectrum of mast-cell disorders.^10^

This phenomenon, called Kounis syndrome, is caused by the enormous activation of mast cells to release systemic inflammatory mediators such as histamine, chemokines, enzymes such as neutral protease chymase, tryptase, cathepsin-D, peptides, proteoglycans, growth factors, and arachidonic acid products that induce the endothelial dysfunction and microthrombus formation in any arterial vessel.^4^,^5^,^10^,^11^ We believe that cerebral blood flow is compromised more significantly than what would be expected from hypotension alone, likely due to the direct action of inflammatory mediators on cerebral vessels.

Recent case reports have shown that the patients’ ischemic brainstem stroke occurred following an allergic reaction triggered by a bee sting, a manifestation potentially linked to Kounis syndrome, a condition characterized by allergic-induced cardiovascular events such as coronary spasm and thrombosis, suggesting its broader involvement in other vascular territories, including cerebral arteries.^6^,^12^ Its link to acute myocardial injury due to allergic reactions, has been increasingly recognized to be a pan-arterial disorder affecting various vascular territories, including coronary, mesenteric, and cerebral arteries.^2^ Although cerebral involvement is rare, the potential exists for allergic reactions to cause transient or permanent neurological deficits, as seen in anaphylaxis-induced stroke, thereby broadening the understanding of anaphylaxis-related complications and underscoring the need for heightened awareness and early intervention.^4^,^12^,^13^ In this case, it was noted that the ECG findings showed ST-segment changes, indicating there may have allergic coronary vessels without experiencing any chest pain. It could be a type I variant of Kounis syndrome, as it resolved after administration of anti-inflammatory agents.^10^

The treatment of cerebral Kounis syndrome is multifaceted and challenging; early intervention is crucial for improving patient outcomes. Epinephrine remains the first-line treatment for anaphylaxis, working rapidly to reverse the effects of vasodilation and bronchospasm.^1^ The patient received intramuscular epinephrine, along with IV antihistamines, corticosteroids, and fluids, which resulted in prompt resolution of both the allergic and neurological symptoms. It suggests that ischemic changes caused by transient hypoperfusion in the context of anaphylaxis, if treated appropriately, may be less severe than those caused by cerebral infarction, where tissue death and irreversible damage are certain.^14^,^15^ The need for a thrombolytic agent may not be necessary as it was noted in one case report that the patient did not improve after therapy.^13^ Other options to prevent cerebral thrombus from becoming unstable are the inhibition of mast-cell degranulation by administering cromolyn and flavonoid quercetin; however, the latter is not commonly available in the hospital setting.^10^

## CONCLUSION

This case report of transient ischemic attack induced by an anaphylactic reaction adds to the growing body of evidence suggesting that anaphylaxis can present with neurological complications, albeit rarely. It emphasizes the need for clinicians to consider a broader differential diagnosis when managing patients presenting with both allergic and neurological symptoms. Prompt recognition and early administration of epinephrine are critical for optimizing outcomes; however, the possibility of an acute ischemic stroke should not be excluded.
